# Adult Patients with Philadelphia-Positive B-Cell Acute Lymphoblastic Leukemia Treated with a Pediatric-Inspired Multiagent Chemotherapy Regimen, in Combination with a TKI, Do Not Require Routine alloSCT

**DOI:** 10.3390/curroncol33020127

**Published:** 2026-02-22

**Authors:** Donna Zhe Sian Eng, Fatima Khadadah, Maria Agustina Perusini, Eshrak Al-Shaibani, Eshetu G. Atenafu, Aniket Bankar, Marta Davidson, Guillaume Richard-Carpentier, Dawn Maze, Karen Yee, Aaron Schimmer, Vikas Gupta, Steven Chan, Dennis Dong Hwan Kim, Andre Schuh, Mark Minden, Hassan Sibai

**Affiliations:** 1 Division of Medical Oncology and Hematology, Princess Margaret Cancer Centre, University Health Network, Toronto, ON M5G 2C4, Canadamark.minden@uhn.ca (M.M.); hassan.sibai@uhn.ca (H.S.); 2 Biostatistics Department, University Health Network, Toronto, ON M5G 1Z5, Canada; 3 Division of Biostatistics, University of Toronto, Toronto, ON M5S 3E3, Canada

**Keywords:** Philadelphia-positive B-cell acute lymphoblastic leukemia, adult, pediatric-inspired chemotherapy

## Abstract

Tyrosine kinase inhibitors (TKIs) added to chemotherapy have improved outcomes of adult patients with Philadelphia-positive B-cell acute lymphoblastic leukemia (Ph+ B-ALL). These improvements initially led to a larger proportion of patients realizing allogeneic stem cell transplantation (alloSCT), long considered essential for cure, but there has been a re-evaluation of alloSCT. At Princess Margaret Hospital (PM), adult patients with Ph+ B-ALL have been treated with a pediatric-inspired chemotherapy protocol with mostly imatinib. In the last two decades, we have witnessed many iterative changes in our approach. Here, we examine the outcomes of all Ph+ B-ALL patients treated at our institution from 2001 to 2019. During this time, there were two major protocol changes—omission of asparaginase in 2009 and discontinuation of routine referral for first complete remission (CR1) alloSCT from the early 2010s. Median follow-up was 41.13 months (range, 0.46–228.79). In total, 141 patients (91.56%) achieved CR1. Patient outcomes improved iteratively, with the best results seen in the final (2016–2019) cohort: no asparaginase, no routine alloSCT referral in CR1; 4-year overall survival (OS) and relapse-free survival (RFS) were 87.0% and 69.3%, respectively. The long-term OS in this patient group retained statistical significance in the multivariable analysis (*p* = 0.0176) when BCR::ABL1 molecular measurable residual disease (MRD) was considered.

## Simple Summary

Tyrosine kinase inhibitors (TKIs) added to the pediatric-inspired Princess Margaret-Dana Farber Cancer Institute (PM-DFCI) chemotherapy protocol demonstrate that omitting asparaginase and routine allogeneic stem cell transplantation (alloSCT) in Philadelphia-positive B-cell acute lymphoblastic leukemia (Ph+ B-ALL) significantly improves survival by reducing excess toxicity, provided that treatment decisions are guided by BCR::ABL1 measurable residual disease.

## 1. Introduction

The treatment landscape of adult Ph+ B-ALL has changed considerably since the addition of TKIs to chemotherapy. At PM, our approach has been to use a pediatric-inspired chemotherapy protocol, Princess Margaret-Dana Farber Cancer Institute (PM-DFCI) (originally derived from DFCI 10-175/NCIC CTG AL.4), combined with a TKI (usually imatinib), and with modifications for ages <60 (PM-DFCI-3.n) and ≥60 (PM-DFCI-4.n) years [1,2,3]. Our approach (Figures S1 and S2) has been adopted by many other institutions in Canada. Increased toxicity observed early on when imatinib was added led to the subsequent omission of asparaginase in 2009. This approach was subsequently adopted by other groups due to toxicity seen in their patients [4,5].

**Figure 1:**
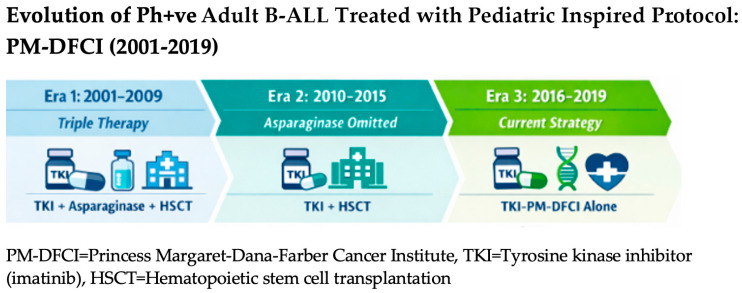
Schema of treatment modifications.

In the early 2010s, there was evidence for better safety and efficacy of chemotherapy/TKI combinations. Further, we observed that many patients for whom we were unable to find an alloSCT donor were nevertheless achieving good long-term outcomes without consolidative alloSCT, and we started to question the value of obligatory transplantation. Our approach evolved further such that only patients with an inadequate MRD response were referred for CR1 alloSCT. Figure 1 illustrates the treatment modifications made over the years. This contrasts with some centres where alloSCT remains the mainstay of consolidative treatment. The European Society for Blood and Marrow Transplantation (EBMT) recommends CR1 alloSCT as the standard of care, regardless of MRD results [6]. However, our approach has been adopted by many other centres, and consistent with this, the National Comprehensive Cancer Network (NCCN) now recommends consolidative alloSCT only for patients with persistent or rising MRD after minimizing MRD with additional treatment [7]. While NCCN 2024 explicitly states that there is no CR1 alloSCT advantage for those ≤21 years if they have achieved MRD negativity, for patients >21 years, the transplant recommendation remains less clear [7]. Additionally, while the American Society for Transplantation and Cellular Therapy recommends CR1 alloSCT for patients receiving TKIs, it remains unclear whether this recommendation should apply to patients achieving complete molecular remission (CMR) [8].

Given the evolution, this retrospective review aims to examine the effectiveness of our approach, with a particular focus on the roles of asparaginase, CR1 alloSCT, and MRD monitoring, on overall survival (OS) and relapse-free survival (RFS), and to compare our outcomes with those reported in the literature.

## 2. Methods

### 2.1. Patients

Between March 2001 and March 2019, 157 patients (≥18 years) with newly diagnosed Ph+ B-ALL were treated at PM. Patients were last followed up on in May 2022. Three patients were excluded due to initial treatment with imatinib/steroid, single-agent imatinib, and amsacrine/vincristine, respectively. In total, 152 patients were treated with our pediatric-inspired multiagent regimen in combination with imatinib and 12 intrathecal chemotherapies. Two additional patients treated with the original NCIC CTG AL.4 or HyperCVAD protocols were included. All patients except one, who received an unknown TKI, received imatinib upfront, while 2 patients received dasatinib after intolerance to imatinib early in their treatment course. After completion of chemotherapy, the TKI was continued indefinitely as maintenance therapy. In total, 141 of the 154 (91.56%) patients achieved CR1. Prior to 2015–2016, patients were universally referred for consolidative alloSCT, regardless of MRD response, if they were appropriate transplant candidates and matched related or unrelated donors were identified. Between 2016 and 2019, however, only eight patients underwent alloSCT in CR1—two due to therapy-related disease, four due to flow cytometric MRD positivity post-induction or at 3 months, one due to a very slow reduction in BCR::ABL1 levels, and another due to patient choice after discussion with the transplant team (in the first year after the decision to shift away from CR1 alloSCT, patient referrals were still being made to facilitate patient informed consent). This treatment review was approved by the University Health Network Ethics Board.

### 2.2. Response Definitions

BCR::ABL1 molecular response criteria used in this analysis are BCR::ABL1:ABL1 ratio ≤0.1% on the International Scale for the p210 transcripts or ≥3-log reduction in p190 transcripts. The standard practice at our institution is to measure BCR::ABL1 messenger ribonucleic acid in the bone marrow post-induction and to follow up with the same testing method every 3 months as well as to allow peripheral blood BCR::ABL1 testing after longer periods of remission when bone marrow sampling shows undetectable results. Relapse was defined by the recurrence of ≥5% blasts in a bone marrow aspirate/biopsy or by the new presence of extramedullary disease. Time to RFS was calculated in months from the date of CR1 until relapse or death. Time to OS was calculated in months from the date of CR1 until death.

### 2.3. Statistical Analysis

Patient characteristics were summarized using median (range) for continuous variables and frequencies (percentages) for categorical variables of interest. Comparisons of baseline characteristics of the two groups (those who underwent CR1 alloSCT vs. those who did not undergo CR1 alloSCT) were made using chi-square or Fisher’s exact tests (as appropriate) for categorical variables and the Student’s *t*-test for continuous variables. RFS and OS rates were calculated using the Kaplan–Meier estimates, and the impact of covariates of interest was assessed using the log-rank test. Multivariable Cox proportional hazards models were used to assess the joint impact of covariates on RFS and OS. All *p*-values were 2-sided. For the statistical analyses, *p* < 0.05 was considered statistically significant. Statistical analysis was performed using version 9.4 of the SAS system for Windows, Copyright © 2023 by SAS Institute, Inc., Cary, NC, USA.

## 3. Results

Patient clinical and demographic characteristics are summarized in Table 1. In total, 141 patients achieved CR1. There were five induction deaths. One patient who did not receive asparaginase died from bacteremia, viremia, electrolyte disturbance, and subdural hemorrhages/infarcts in the context of CNS disease. The other four patients had received asparaginase—two of them died from liver failure, pneumonia, and upper gastrointestinal bleeding (one also with methotrexate toxicity); one died from liver and renal failure in the context of septicemia from Aspergillus; and another patient died from cardiac, renal failure, and pneumonia.

Median follow-up was 41.13 months (range, 0.46–228.79). Fifty-three (37.59%) patients underwent CR1 alloSCT. These patients, and those who did not undergo CR1 alloSCT, had similar baseline characteristics with regard to median white blood cell (WBC) count, prior cancer diagnosis, extramedullary disease, and central nervous system (CNS) involvement. The only difference between these two groups was patient age, with the transplanted cohort being significantly younger (median, 46 vs. 55 years; *p* < 0.001). These patient characteristics are outlined in Table S2. Median time from diagnosis to CR1 was 1.18 months (range, 0.66–2.89). The median time from CR1 to alloSCT in CR1 was 6.97 months (range, 1.91–20.52). Twenty-five patients had matched sibling donors, 39 had matched unrelated donors, and one patient had a cord blood donor, while information on the donor was missing for one patient. Patients continued receiving TKI maintenance up until alloSCT. Only 12 patients received TKI maintenance post-alloSCT, as its role was unclear at the time when these transplants were performed.

From 2001 to 2019, thirty-five (24.8%) patients received asparaginase. In 2001–2009, all 35 patients received asparaginase. After 2009, no patients received asparaginase.

### 3.1. Post-Induction Status and Survival

**Figure 2:**
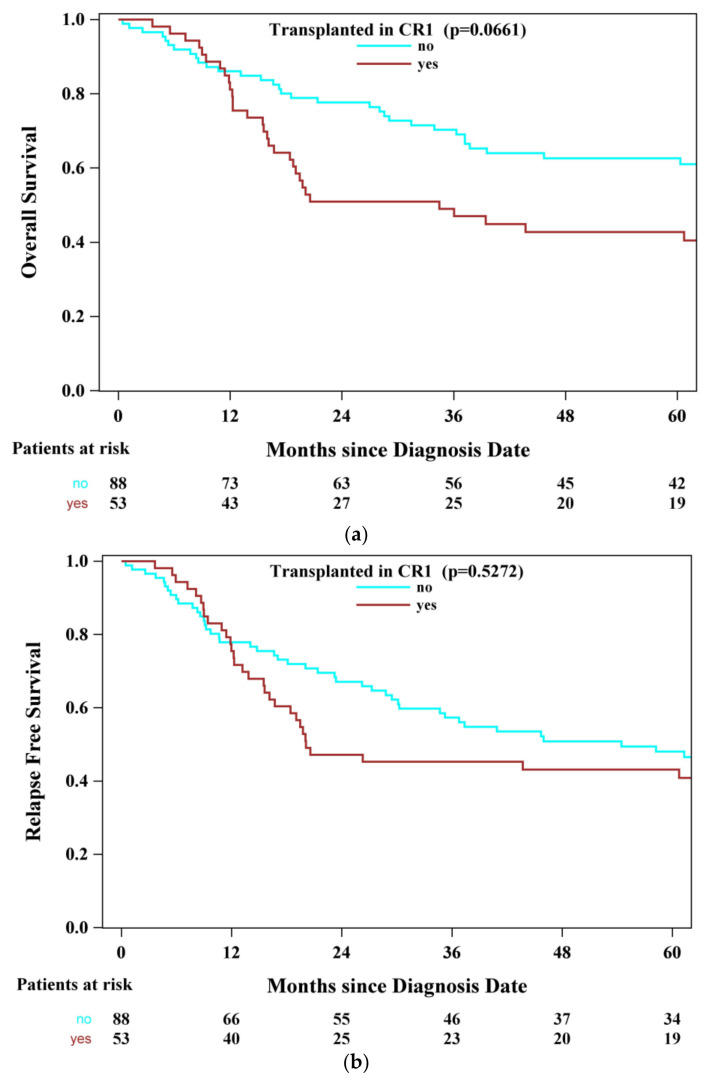
(**a**) Overall survival stratified by alloHSCT in CR1 and (**b**) RFS stratified by alloHSCT in CR1.

For the entire cohort, 4-year OS was 54.9% (95% CI, 46.1–62.9%) and 4-year RFS was 47.8% (95% CI, 39.2–56.0%). Four-year OS for patients who underwent CR1 alloSCT was 42.7% (95% CI, 29.2–55.6%), compared to 62.6% (95% CI, 51.2–72.1%) for those who received chemotherapy and imatinib alone (Figure 2a). There was a trend towards improved OS, but overall, there was no statistical difference (*p* = 0.0661). RFS (Figure 2b), though, did not differ significantly between the two groups—4-year RFS was 43.1% (95% CI, 29.6–55.9%) and 50.9% (95% CI, 39.6–61.0%) (*p* = 0.5272) with and without alloSCT, respectively.

**Figure 3:**
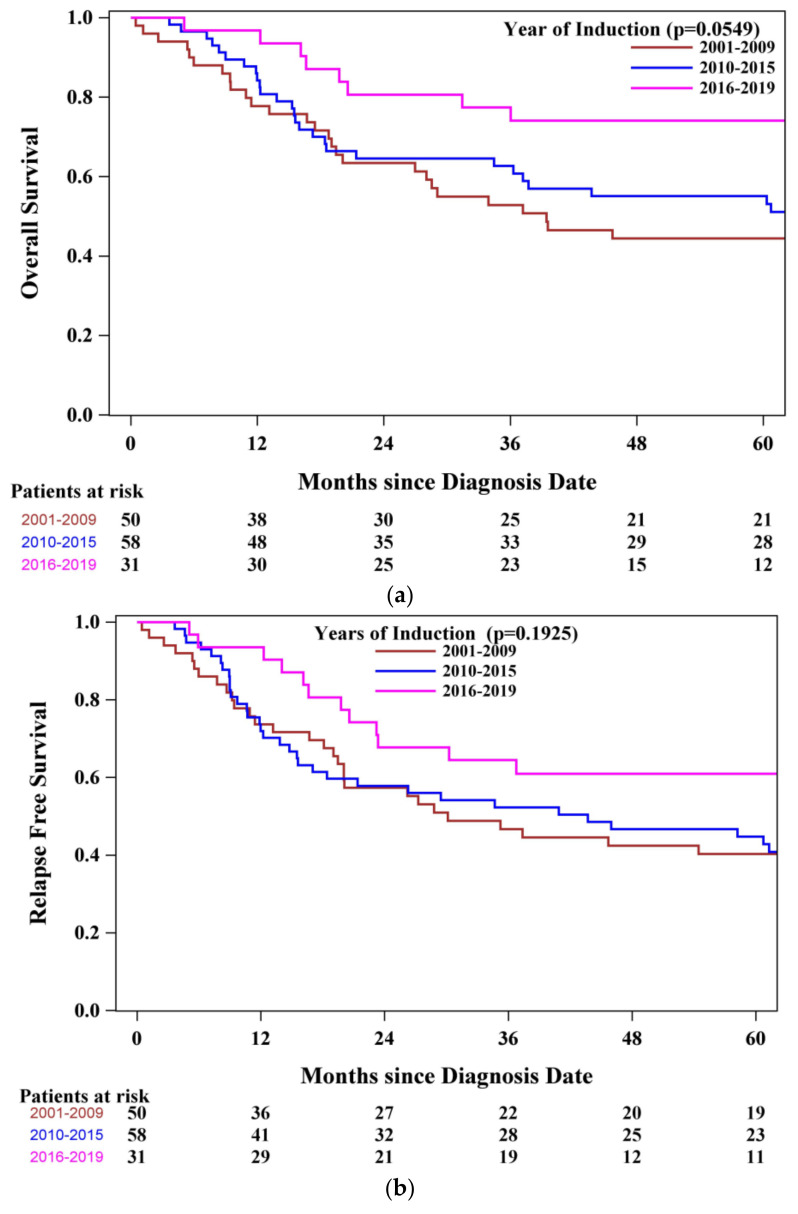
(**a**) Overall survival according to year of induction. (**b**) Relapse-free survival according to year of induction.

The first protocol amendment was the removal of asparaginase after 2009. From 2015, another significant modification was a shift away from CR1 alloSCT. To assess how these changes may have influenced outcomes over time, we grouped patients into three cohorts according to induction treatment time period: 2001–2009, 2010–2015, and 2016–2019. There were 50, 58, and 31 patients, respectively. Unsurprisingly, patients who were treated in the most recent years, 2016–2019, demonstrated a significantly improved OS (HR of 0.394 [95% CI, 0.180–0.864]), while patients treated in 2010–2015 were not significantly different from patients treated in 2001–2009 (HR 0.873 [95% CI, 0.528–1.443]), *p* = 0.0201 and *p* = 0.5970, respectively (Figure 3). These results were not influenced by the length of follow-up—median follow-up of 47.84 months (range, 5.06–73.35), 50.45 months (range, 3.65–141.83), and 35.56 months (range, 0.46–228.20), respectively. Although there appears to be a trend towards longer RFS in the more recent groups, this did not reach statistical significance—HR of 0.571 (95% CI, 0.292–1.116, *p* = 0.1012) and HR of 1.008 (95% CI, 0.623–1.630, *p* = 0.9747) were detected, respectively.

**Figure 4:**
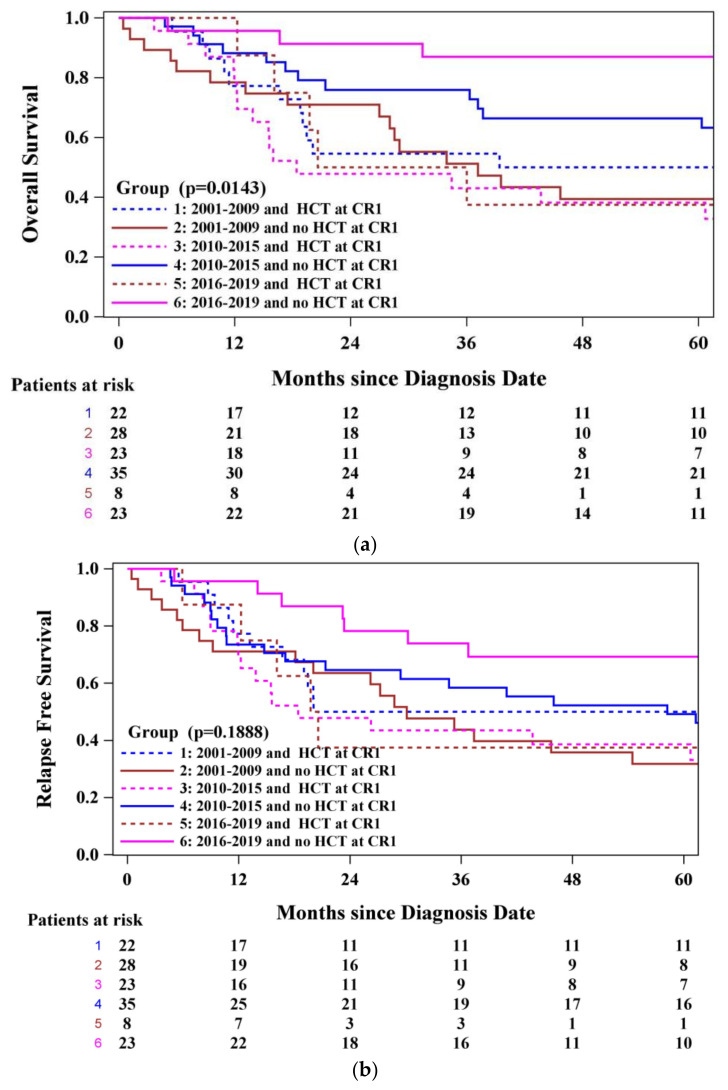
(**a**) Overall survival according to year of induction and alloSCT in CR1. (**b**) RFS according to year of induction and alloSCT in CR1.

When alloSCT in CR1 and year of induction were considered together, the 23 patients diagnosed between 2016 and 2019 who did not undergo CR1 alloSCT had the best outcomes—4-year OS of 87.0% (95% CI, 64.8–95.6) (*p* = 0.0143) and 4-year RFS of 69.3% (95% CI, 46.1–84.0) (*p* = 0.1888) (Figure 4). Patients who underwent induction in 2001–2009, whether they underwent (22 patients) or did not undergo (28 patients) transplantation, had outcomes that were similarly unfavourable—4-year OS of 50.0% (95% CI, 28.2–68.4) versus 39.4% (95% CI, 21.1–57.3), and 4-year RFS of 50.0% (95% CI, 28.2–68.4) versus 35.8% (95% CI, 18.3–53.7), respectively. Intermediate outcomes were observed in the 35 patients who commenced therapy in 2010–2015 and did not undergo consolidative alloSCT—4-year OS of 66.5% (95% CI, 47.6–79.9) and 4-year RFS of 52.3% (95 CI, 34.3–67.5). These results underscore that omitting asparaginase did not have a detrimental impact on RFS. See Supplementary Table S1 for a full analysis according to year of induction stratified by alloSCT in CR1.

### 3.2. Univariate and Multivariable Analyses

Age <60 and ≥60 years, sex, WBC at presentation, extramedullary disease and CNS involvement, prior cancer, asparaginase use, alloSCT as a time-varying variable, years of induction, and BCR::ABL1 MRD results at 3/6 months and 9/12 months (<3-log reduction and ≥3-log reduction in transcripts) were identified as important factors for univariate analysis (Table 2). Patients with ≥3-log reduction in BCR::ABL1 transcripts at 9/12 months had significantly longer OS and RFS than patients with <3-log reduction (HR of 0.35 [95% CI, 0.16–0.79]; *p* = 0.0115 and HR of 0.38 [95% CI, 0.18–0.81]; *p* = 0.0120), respectively. Notably, ≥3-log reduction in BCR::ABL1 transcripts at earlier timepoints (3/6 months) was not statistically significant for either OS or RFS. Interestingly, alloSCT as a time variable was demonstrated to result in statistically inferior OS and RFS (HR 2.19 [95% CI, 1.35–3.57]; *p* = 0.0016) and (HR 1.73 [95% CI, 1.09–2.74]; *p* = 0.0204), respectively. Asparaginase use was not significantly associated with inferior outcomes, though this could be due to small patient numbers.

In the multivariable model (Table 3), ≥3-log reduction in BCR::ABL1 transcripts at 9/12 months and alloSCT as a time variable retained statistical significance for OS and RFS. BCR::ABL1 transcript at 9/12 months had an HR of 0.37 [95% CI, 0.15–0.94]; *p* = 0.0368 for OS and HR of 0.36 [95% CI, 0.15–0.86]; *p* = 0.0220) for RFS, respectively, while alloSCT as a time-varying variable had HR of 2.26 [95% CI, 1.18–4.33]; *p* = 0.0139) for OS and HR of 1.88 [95% CI, 1.01–3.48]; *p* = 0.0452) for RFS, respectively. When examining the different cohorts according to induction year, there was no statistical difference among patients treated during any of these separate timepoints.

## 4. Discussion

Adult patients with newly diagnosed Ph+ B-ALL have long been treated with chemotherapy and TKI. Recent reports, including the D-ALBA study and preliminary results from the phase 3 GIMEMA ALL2820 trial, have demonstrated that frontline immunotherapy, in particular using blinatumomab, can lead to better outcomes [9,10,11]. Nevertheless, chemotherapy backbones remain the standard of care in most centres. We, and several other Canadian institutions, use pediatric-inspired multiagent chemotherapy protocols in combination with imatinib. Though consolidative alloSCT continues to be performed routinely in many centres and is still recommended in some guidelines, such as those by EBMT [6], we have shifted away from CR1 alloSCT. The evolution of our practice—omission of asparaginase and alloSCT in CR1—was based on excess toxicity and inferior outcomes observed in earlier years. This study examined whether our protocol changes addressed these concerns.

The efficacy of our approach appears to be at least comparable to the literature. The EBMT registry reported that patients with Ph+ B-ALL transplanted in CR1 between 2016 and 2020 had 3-year OS of 73% and 77% if MRD-positive or MRD-negative, respectively, pre-transplant [12]. Our patients who received induction in 2016–2019 and did not undergo CR1 alloSCT demonstrated a superior 4-year OS of 87.0% (95% CI, 64.8.1–95.6). In fact, the multivariable analysis here revealed that alloSCT in CR1 as a time-dependent variable negatively affected both OS and RFS. This supports the notion that universal CR1 alloSCT is not indicated even when the TKI used is imatinib, in contrast to prior suggestions that CR1 alloSCT improves long-term survival rates when first- or second-generation TKIs are used [13,14,15].

Stratifying our patients by time period helped address potentially confounding issues, such as improvements in supportive care, and facilitated the comparison with time-matched cohorts from the literature. Focusing first on our initial approach that incorporated asparaginase and alloSCT, only the UKALLXII protocol reported by Fielding et al. (2014) used a pediatric-inspired multiagent chemotherapy protocol that also incorporated L-asparaginase [16]. Other contemporaneous adult studies utilizing chemotherapy and imatinib largely used conventional adult-type protocols [4,14,17,18,19]. The former delivered more intensive chemotherapy followed by prolonged maintenance chemotherapy. The UKALLXII trial transplanted a substantial number (72%) of patients, resulting in 4-year OS and RFS of 38% and 50%, respectively [16]. Our patients were induced in 2001–2009, of whom 44.0% received consolidative alloSCT; 4-year OS and RFS were 44.4% (95% CI, 30.2–57.7%) and 42.5% (28.4–55.8%), respectively.

Asparaginase toxicity in our cohort is also comparable to that observed in UKALLXII and later studies. A retrospective analysis of our patients when asparaginase was part of the protocol found that most toxicities occurred during induction or intensification phases, with asparaginase being held or discontinued largely due to liver function abnormalities [2]. Grade 3–4 toxicities were up to 38% during induction and 54% during intensification phases of treatment [2]. Due to toxicities, only six of 28 patients (21%) who entered the intensification phase received ≥80% of the intended cumulative dose of asparaginase [2]. In this report, four out of five patients who died during induction had received asparaginase after TKI was added to the protocol. While this observation does not necessarily indicate causality, it is consistent with the results of the UKALL14 trial that recruited patients between 2010 and 2012, which added pegylated-asparaginase and imatinib to their pediatric-inspired intensive induction regimen [5]. Ph+ patients had a >8-fold increase in induction deaths as compared to Ph- disease, which remained significant in the multivariable analysis [5]. Consequently, pegylated-asparaginase was removed from the UKALL14 induction protocol for patients with Ph+ B-ALL [5]. Asparaginase is unlikely to cause late toxicities, as Thyagu and colleagues (2012) showed that patients who had a matched related or unrelated donor and underwent transplantation had a non-significant 3-year EFS of 47% versus 73% for the non-donor group (HR 1.08, 95% CI, 0.38–3.05, *p* = 0.88) and similar 3-year OS of 56% and 50% (HR 0.58, 95% CI, 0.19–1.79, *p* = 0.34), respectively [2].

Univariate and multivariable analyses here demonstrate that OS and RFS can be predicted more precisely by BCR::ABL1 MRD monitoring. Achieving ≥3-log reduction at 9 and 12 months correlated with significantly better OS (HR of 0.351 [95% CI, 0.156–0.791], *p* = 0.0115) and RFS (HR of 0.381 [95% CI, 0.180–0.809], *p* = 0.0120). A 3-log reduction was chosen as the benchmark response because this was previously adopted by different groups, including Ravandi et al. (2013) and Chalandon et al. (2015), as an appropriate molecular response criterion [20,21]. Notably, many of our patients have been observed to have longstanding detectable BCR::ABL1 transcripts, at levels ≥3-log reduction, with no signs of relapse [20,21]. Ravandi et al. (2013) furthermore found no difference in OS when comparing patients attaining MMR and CMR at 3, 6, 9, and 12 months, who were treated with hyperCVAD and imatinib or dasatinib, and no consolidative alloSCT [20]. Ravandi et al. (2013), however, did not report RFS that would have corroborated our findings [20].

The literature is clear that newer-generation TKIs can achieve deeper molecular responses faster, with ponatinib having the highest 3-month CMR rate [22,23]. A meta-analysis by Jabbour et al. (2018) comparing one study using HyperCVAD and ponatinib with pooled results from 25 studies using first- and second-generation TKI/chemotherapy combinations reported greater CMR and 3-year OS rates of 79% and 79%, versus 34% and 50%, respectively, favouring ponatinib [24]. Jabbour et al. (2018) further suggested that using ponatinib may eliminate the need for consolidative alloSCT [24]. Nevertheless, the results from this meta-analysis need to be interpreted with caution due to significant heterogeneity among studies and because the comparison was made to a single ponatinib study. Although ponatinib was associated with the highest 3-month CMR rate, Badar et al. (2023b) did not find any differences in RFS among non-transplant patients reaching CMR at 3 months with various TKIs compared to those who did not achieve CMR, although OS was statistically superior in the former group [22]. Short et al. (2016) also demonstrated that while ponatinib and HyperCVAD without consolidative alloSCT induced deep molecular responses in a greater number of patients, outcomes did not differ when similarly deep responses were achieved using imatinib or dasatinib [23]. Among their patients, 4-year OS and RFS for patients achieving CMR by 3 months were 66% and 63%, respectively, which is inferior to the results reported here, possibly reflecting different chemotherapy backbones [23].

We do not have information on molecular mutations in our patients; however, not achieving ≥3-log reduction at 9 and 12 months may indicate poor-risk disease biology, as reflected by IKZF1 deletion alone or additional genetic aberrations including CDKN2A, CDKN2B, PAX5, or both (IKZF^plus^). Conventional chemotherapy and imatinib may be inadequate in such patients [13,25,26]. Based on our data, we show that integrating BCR::ABL1 MRD monitoring allows a decision to be made about omitting alloSCT in CR1. Therefore, patients who do not achieve ≥3-log reduction at 9 and 12 months would benefit from alternative approaches, including pre-emptive or targeted (if ABL kinase domain mutations are found) switches to newer-generation TKIs, use of targeted chemotherapy, i.e., inotuzumab, and immunotherapy, i.e., blinatumomab or chimeric antigen receptor T-cell therapy, and should be considered for consolidative alloSCT. If alloSCT is considered, it makes sense to deepen MRD prior to the procedure. In the phase 2 D-ALBA study, the adverse disease biology of IKZF1/IKZF1^plus^ could be overcome with blinatumomab consolidation post pre-phase glucocorticoids and dasatinib induction [25]. We await further analysis of the GIMEMA ALL2820 phase 3 trial comparing chemotherapy and imatinib with ponatinib and blinatumomab, which has demonstrated early encouraging 18-month event-free survival of 76.8% versus 89.9% (*p* = 0.011), respectively [9].

Our study’s main limitation is the bias inherent to retrospective analyses. By stratifying patients according to the time periods during which asparaginase and CR1 alloSCT were omitted from our protocol and by analyzing the specific impact of these changes using univariate and multivariable analyses, potential selection bias and unknown confounders were minimized. In addition, the lack of further cytogenetic and molecular information limits further correlation with our findings.

## 5. Conclusions

Our analysis is instructive in several regards. While the treatment paradigm is evolving with the incorporation of newer therapeutic agents at earlier timepoints, our data indicate that our pediatric-inspired multi-agent chemotherapy backbone results in excellent outcomes, even if imatinib is used. Our data further support the notion that omission of asparaginase in Ph+ B-ALL is associated with better outcomes, and CR1 alloSCT is not obligatory as long as MRD targets are met.

## Figures and Tables

**Table 1:** Patient characteristics and treatment regimens.

Parameter	All Patients *N* = 141	2002–2009 *N* = 50	2010–2015 *N* = 58	2016–2019 *N* = 31
Median age, year (range)	50.00 (18.0–79.1)	49.00 (18.0–78.4)	50.00 (21.4–79.1)	53.00 (24.0–72.6)
Age ≥ 60, *n* (%)	39 (27.66)	13 (26.00)	16 (27.59)	9 (29.03)
Male, *n* (%)	77 (54.61)	25 (50.00)	35 (60.34)	16 (51.61)
Female, *n* (%)	64 (45.39)	25 (50.00)	23 (39.66)	15 (48.39)
Median WBC count, ×10^9^/L (range)	19.20 (0.80–272.0)	19.00 (0.80–140.0)	24.00 (0.90–272.0)	14.00 (1.7–82.6)
WBC count, ≥30 ×10^9^/L (%)	45 (34.35)	15 (32.61)	23 (41.82)	7 (23.33)
Extramedullary disease, *n* (%)	14 (9.93)	7 (14.00)	4 (6.90)	3 (9.68)
CNS involvement at diagnosis, *n* (%)	15 (10.64)	10 (20.00)	3 (5.17)	2 (6.45)
Prior cancer history, n (%)	13 (9.63)	4 (8.00)	5 (8.77)	4 (15.38)
**Chemotherapy, *n* (%)**				
Pediatric-inspired multiagent protocol	140 (99.29)	50 (100.00)	58 (100.00)	30 (96.77)
Other	1 (0.71)	0 (0.00)	0 (0.00)	1 (3.23)
Asparaginase, *n* (%)	35 (24.82)	35 (70.00)	0 (0.00)	0 (0.00)
**TKI, *n* (%)**				
Imatinib	138 (98.57)	48 (97.96)	57 (98.28)	31 (100.00)
Dasatinib	2 (1.43)	1 (2.04)	1 (1.72)	0 (0.00)
**Transplant in CR1, *n* (%)**	53 (37.59)	22 (44.00)	23 (39.66)	8 (25.81)

**Table 2:** Results from univariate analysis.

Variable	OS			RFS		
	HR	95% CI	*p*-Value	HR	95% CI	*p*-Value
Age (≥60 years)	0.91	(0.54, 1.54)	0.7255	1.06	(0.65, 1.72)	0.8198
Sex (female)	1.52	(0.95, 2.43)	0.0824	1.42	(0.92, 2.20)	0.1162
WBC (≥30 ×10^9^/L)	1.27	(0.78, 2.10)	0.3389	1.24	(0.78, 1.97	0.3696
Extramedullary disease + CNS involvement (present)	1.20	(0.67, 2.17)	0.5434	0.95	(0.53, 1.70)	0.8690
Prior cancer (present)	1.51	(0.72, 3.18)	0.2720	1.69	(0.84, 3.41)	0.1384
Asparaginase (yes)	1.10	(0.65, 1.87)	0.7245	0.99	(0.60, 1.65)	0.9824
Year of induction (ref = 2001–2009)	0.87	(0.53, 1.44)	0.0651 0.5970	1.01	(0.62, 1.63)	0.2008 0.9747
2010–2015						
2016–2019	0.39	(0.18, 0.86)	0.0201	0.57	(0.29, 1.12)	0.1012
HCTCR1t (time varying)	2.19	(1.35, 3.57)	0.0016	1.73	(1.09, 2.74)	0.0204
BCR::ABL1 3/6 months (≥3-log reduction)	1.09	(0.51, 2.32)	0.8329	1.02	(0.51, 2.03)	0.9557
BCR::ABL1 9/12 months (≥3-log reduction)	0.35	(0.16, 0.79)	0.0115	0.38	(0.18, 0.81)	0.0120

**Table 3:** Results from multivariable analysis.

Variable	OS			RFS		
	HR	95% CI	*p*-Value	HR	95% CI	*p*-Value
Sex (female)	1.66	(0.90, 3.06)	0.1016	1.53	(0.87, 2.69)	0.1371
Prior cancer (present)	1.35	(0.52, 3.56)	0.5385	1.56	(0.65, 3.74)	0.3215
BCR::ABL1 9/12 months (≥3-log reduction)	0.37	(0.15, 0.94)	0.0368	0.36	(0.15, 0.86)	0.0220
Year of induction (ref = 2001–2009)			0.2876			0.2870
2010–2015	1.24	(0.63, 2.45)	0.5350	1.39	(0.74, 2.62)	0.3021
2016–2019	0.47	(0.13, 1.73)	0.2583	0.71	(0.25, 2.03)	0.5211
HCTCR1t (time varying)	2.26	(1.18, 4.33)	0.0139	1.88	(1.01, 3.48)	0.0452

## Data Availability

The data presented in this study are available on request from the corresponding author.

## Supplementary Materials

The following supporting information can be downloaded at https://www.mdpi.com/article/10.3390/curroncol33020127/s1: Figure S1: PM-DFCI-3.n pediatric-inspired multiagent chemotherapy protocol for adult Ph+ B-ALL patients with age under 60; Figure S2: PM-DFCI-4.n pediatric-inspired multiagent chemotherapy protocol for adult Ph + ve ALL patients with age above 60; Table S1: 48-month OS and RFS according to year of induction stratified by alloSCT in CR1; Table S2: Characteristics of patients who underwent alloSCT in CR1 and those who did not.

## References

[1] Martell M.P., Atenafu E.G., Minden M.D., Schuh A.C., Yee K.W.L., Schimmer A.D., Gupta V., Brandwein J.M. (2013). Treatment of elderly patients with acute lymphoblastic leukaemia using a paediatric-based protocol. Br. J. Haematol..

[2] Thyagu S., Minden M.D., Gupta V., Yee K.W., Schimmer A.D., Schuh A.C., Lipton J.H., Messner H.A., Xu W., Brandwein J.M. (2012). Treatment of Philadelphia chromosome-positive acute lymphoblastic leukaemia with imatinib combined with a paediatric-based protocol. Br. J. Haematol..

[3] Kanfar S.S., Chan S.M., Gupta V., Schimmer A.D., Schuh A.C., Sibai H., Yee K.W., Minden M.D. (2016). Outcomes of Adult Philadelphia Positive Acute Lymphoblastic Leukemia Patients Treated with Pediatric Multi-Agent Chemotherapy and Imatinib and the Impact of Residual Disease Monitoring on Survival. Blood.

[4] Bassan R., Rossi G., Pogliani E.M., Di Bona E., Angelucci E., Cavattoni I., Lambertenghi-Deliliers G., Mannelli F., Levis A., Ciceri F. (2010). Chemotherapy-Phased Imatinib Pulses Improve Long-Term Outcome of Adult Patients with Philadelphia Chromosome-Positive Acute Lymphoblastic Leukemia: Northern Italy Leukemia Group Protocol 09/00. J. Clin. Oncol..

[5] Patel B., A Kirkwood A., Dey A., I Marks D., McMillan A.K., Menne T.F., Micklewright L., Patrick P., Purnell S., Rowntree C.J. (2017). Pegylated-asparaginase during induction therapy for adult acute lymphoblastic leukaemia: Toxicity data from the UKALL14 trial. Leukemia.

[6] Snowden J.A., Sánchez-Ortega I., Corbacioglu S., Basak G.W., Chabannon C., de la Camara R., Dolstra H., Duarte R.F., Glass B., Greco R. (2022). Indications for haematopoietic cell transplantation for haematological diseases, solid tumours and immune disorders: Current practice in Europe, 2022. Bone Marrow Transplant..

[7] Shah B., Mattison R.J., Abboud R., Abdelmessieh P., Aldoss I., Burke P.W., DeAngelo D.J., Dinner S., Fathi A.T., Gauthier J. (2024). Acute Lymphoblastic Leukemia, Version 2.2024, NCCN Clinical Practice Guidelines in Oncology. J. Natl. Compr. Cancer Netw..

[8] DeFilipp Z., Advani A.S., Bachanova V., Cassaday R.D., Deangelo D.J., Kebriaei P., Rowe J.M., Seftel M.D., Stock W., Tallman M.S. (2019). Hematopoietic Cell Transplantation in the Treatment of Adult Acute Lymphoblastic Leukemia: Updated 2019 Evidence-Based Review from the American Society for Transplantation and Cellular Therapy. Biol. Blood Marrow Transplant..

[9] Chiaretti S., Di Trani M., Skert C., Elia L., Almici G., Della Starza I., Cardinali D., Bellomarino V., Soddu S., Messina M. (2025). First results of the Phase III GIMEMA ALL2820 trial comparing ponatinib plus blinatumomab to imatinib and chemotherapy for newly diagnosed adult ph+ acute lymphoblastic leukemia patients. Blood.

[10] Foà R., Bassan R., Elia L., Piciocchi A., Soddu S., Messina M., Ferrara F., Lunghi M., Mulè A., Bonifacio M. (2024). Long-Term Results of the Dasatinib-Blinatumomab Protocol for Adult Philadelphia-Positive ALL. J. Clin. Oncol..

[11] Jabbour E., Short N.J., Jain N., Huang X., Montalban-Bravo G., Banerjee P., Rezvani K., Jiang X., Kim K.H., Kanagal-Shamanna R. (2023). Ponatinib and blinatumomab for Philadelphia chromosome-positive acute lymphoblastic leukaemia: A US, single-centre, single-arm, phase 2 trial. Lancet Haematol..

[12] Bazarbachi A., Labopin M., Dalle I.A., Yakoub-Agha I., Socié G., Schroeder T., Blaise D., Poiré X., Balsat M., Salmenniemi U. (2025). Improved post-transplant outcomes since 2000 for Ph-positive acute lymphoblastic leukemia in first remission: A study from the EBMT Acute Leukemia Working Party. HemaSphere.

[13] Badar T., Alkhateeb H., Aljurf M., Kharfan-Dabaja M.A. (2023). Management of Philadelphia chromosome positive acute lymphoblastic leukemia in the current era. Curr. Res. Transl. Med..

[14] Ribera J.M., Ribera J., Genescà E. (2018). The role of stem cell transplantation in the management of Philadelphia chromosome-positive acute lymphoblastic leukemia. Ther. Adv. Hematol..

[15] Saleh K., Fernandez A., Pasquier F. (2022). Treatment of Philadelphia Chromosome-Positive Acute Lymphoblastic Leukemia in Adults. Cancers.

[16] Fielding A.K., Rowe J.M., Buck G., Foroni L., Gerrard G., Litzow M.R., Lazarus H., Luger S.M., Marks D.I., McMillan A.K. (2014). UKALLXII/ECOG2993: Addition of imatinib to a standard treatment regimen enhances long-term outcomes in Philadelphia positive acute lymphoblastic leukemia. Blood.

[17] Lee K.-H., Lee J.-H., Choi S.-J., Seol M., Lee Y.-S., Kim W.-K., Lee J.-S., Seo E.-J., Jang S., Park C.-J. (2005). Clinical effect of imatinib added to intensive combination chemotherapy for newly diagnosed Philadelphia chromosome-positive acute lymphoblastic leukemia. Leukemia.

[18] Yanada M., Takeuchi J., Sugiura I., Akiyama H., Usui N., Yagasaki F., Kobayashi T., Ueda Y., Takeuchi M., Miyawaki S. (2006). High complete remission rate and promising outcome by combination of imatinib and chemotherapy for newly diagnosed BCR-ABL-positive acute lymphoblastic leukemia: A phase II study by the Japan Adult Leukemia Study Group. J. Clin. Oncol..

[19] Daver N., Thomas D., Ravandi F., Cortes J., Garris R., Jabbour E., Garcia-Manero G., Borthakur G., Kadia T., Rytting M. (2015). Final report of a phase II study of imatinib mesylate with hyper-CVAD for the front-line treatment of adult patients with Philadelphia chromosome-positive acute lymphoblastic leukemia. Haematologica.

[20] Ravandi F., Jorgensen J.L., Thomas D.A., O’brien S., Garris R., Faderl S., Huang X., Wen S., Burger J.A., Ferrajoli A. (2013). Detection of MRD may predict the outcome of patients with Philadelphia chromosome–positive ALL treated with tyrosine kinase inhibitors plus chemotherapy. Blood.

[21] Chalandon Y., Thomas X., Hayette S., Cayuela J.-M., Abbal C., Huguet F., Raffoux E., Leguay T., Rousselot P., Lepretre S. (2015). Randomized study of reduced-intensity chemotherapy combined with imatinib in adults with Ph-positive acute lymphoblastic leukemia. Blood.

[22] Badar T., Narra R., Mims A., Shallis R.M., Correia G.S.D.C., Hunter C., Kota V.K., Desai S., Patel A.A., Duvall A.S. (2023). Achievement of Undetectable BCR::ABL1 (uBCR::ABL1) Is Predictive of Improved Survival in Philadelphia Chromosome Positive (Ph+ve) Acute Lymphoblastic Leukemia (ALL) Patients Not Receiving Allogeneic Stem Cell Transplantation. Blood.

[23] Short N.J., Jabbour E., Sasaki K., Patel K., O’Brien S.M., Cortes J.E., Garris R., Issa G.C., Garcia-Manero G., Luthra R. (2016). Impact of complete molecular response on survival in patients with Philadelphia chromosome-positive acute lymphoblastic leukemia. Blood.

[24] Jabbour E., DerSarkissian M., Duh M.S., McCormick N., Cheng W.Y., McGarry L.J., Souroutzidis A., Huang H., O’bRien S., Ravandi F. (2018). Efficacy of Ponatinib Versus Earlier Generation Tyrosine Kinase Inhibitors for Front-line Treatment of Newly Diagnosed Philadelphia-positive Acute Lymphoblastic Leukemia. Clin. Lymphoma Myeloma Leuk..

[25] Foà R., Bassan R., Vitale A., Elia L., Piciocchi A., Puzzolo M.-C., Canichella M., Viero P., Ferrara F., Lunghi M. (2020). Dasatinib–Blinatumomab for Ph-Positive Acute Lymphoblastic Leukemia in Adults. N. Engl. J. Med..

[26] Martinelli G., Iacobucci I., Storlazzi C.T., Vignetti M., Paoloni F., Cilloni D., Soverini S., Vitale A., Chiaretti S., Cimino G. (2009). IKZF1 (Ikaros) deletions in BCR-ABL1-positive acute lymphoblastic leukemia are associated with short disease-free survival and high rate of cumulative incidence of relapse: A GIMEMA AL WP report. J. Clin. Oncol..

