# Supplementary material for: Adult Patients with Philadelphia-Positive B-Cell Acute Lymphoblastic Leukemia Treated with a Pediatric-Inspired Multiagent Chemotherapy Regimen, in Combination with a TKI, Do Not Require Routine alloSCT
Source: Curr Oncol. 2026 Feb 22;33(2):127. doi: 10.3390/curroncol33020127 (PMC12939716; doi:10.3390/curroncol33020127)
Supplement: Supplementary file 1 [file curroncol-33-00127-s001.zip › curroncol-4111685-supplementary.pdf]

**Supplement**

**Figure S1. PM-DFCI-3.n pediatric-inspired multiagent chemotherapy protocol for adult Ph+ B-ALL patients with age under 60.**

**Induction**

31 days

- Imatinib 400mg PO daily (continuous)
- Vincristine 2mg IV or Vinblastine 10mg IV (days 1 & 8)
- Vinblastine 10mg IV (day 15)
- Doxorubicin 30mg/$m^2$ IV (day 1 & 2) (LVEF≥50%)
- Methotrexate 4g/$m^2$ IV (day 3)
- Leucovorin 200mg/$m^2$ at 36 hours after start of methotrexate, then 24mg/$m^2$ IV Q6H until methotrexate level <0.1μM
- Prednisolone 10mg/$m^2$ PO QID or Methylprednisolone 8mg/$m^2$ IV QID (days 1-29)
- Intrathecal methotrexate 12mg/cytarabine 40mg/hydrocortisone 15mg (days 15 & 29)

**CNS phase**

21 days

- Imatinib 400mg PO daily (continuous)
- Vinblastine 10mg IV (day 1)
- Doxorubicin 30mg/$m^2$ IV (day 1) (LVEF≥50%)
- 6-mercaptopurine 50mg/$m^2$ PO (day 1-14)
- Intrathecal methotrexate 12mg/cytarabine 40mg/hydrocortisone 15mg (days 1, 4, 8, 11)

**Intensification phase**

21 weeks; seven 3-week cycles

- Imatinib 400mg PO daily (continuous)
- Vinblastine 10mg IV (day 1)
- Doxorubicin 30mg/$m^2$ IV (day 1) (LVEF≥50%)
- 6-mercaptopurine 50mg/$m^2$ PO (day 1-14)
- Dexamethasone 9mg/$m^2$ PO BID (days 1-5)
- Intrathecal methotrexate 12mg/cytarabine 40mg/hydrocortisone 15mg (cycle 6)

**Maintenance phase**

72 weeks; 24 3-week cycles

- Imatinib 400mg PO daily (continuous)
- Vinblastine 10mg IV (day 1)
- 6-mercaptopurine 50mg/$m^2$ PO (day 1-14)
- Dexamethasone 6mg/$m^2$ PO BID (days 1-5)
- Methotrexate 30mg/$m^2$ IV or IM (days 1, 8, 15)
- Intrathecal methotrexate 12mg/cytarabine 40mg/hydrocortisone (cycles, 5, 11, 17 & 23)

**Figure S2. PM-DFCI-4.n pediatric-inspired multiagent chemotherapy protocol for adult Ph+ve ALL patients with age above 60**

**Induction**

31 days

- Imatinib 400mg PO daily (continuous)
- Vincristine 2mg IV or Vinblastine 10mg IV (days 1 & 8)
- Vinblastine 10mg IV (day 15)
- Doxorubicin 30mg/m$^2$ IV (day 1 & 2) (LVEF≥50%)
- Methotrexate 40mg/m$^2$ IV (day 3)
- Leucovorin 15mg IV Q6H x4 doses
- Methylprednisolone 210mg/m$^2$ IV daily (days 1-4, 9-12)
- Intrathecal methotrexate 12mg/cytarabine 40mg/hydrocortisone 15mg (days 15 & 29)

↓

**CNS phase**

21 days

- Imatinib 400mg PO daily (continuous)
- Vinblastine 10mg IV (day 1)
- Doxorubicin 30mg/m$^2$ IV (day 1) (LVEF≥50%)
- 6-mercaptopurine 50mg/m$^2$ PO (day 1-14)
- Intrathecal methotrexate 12mg/cytarabine 40mg/hydrocortisone 15mg (days 1, 4, 8, 11)

↓

**Intensification phase**

21 weeks; seven 3-week cycles

- Imatinib 400mg PO daily (continuous)
- Vinblastine 10mg IV (day 1)
- Doxorubicin 30mg/m$^2$ IV (day 1) (LVEF≥50%)
- 6-mercaptopurine 50mg/m$^2$ PO (day 1-14)
- Dexamethasone 6mg PO BID (days 1-5)
- Intrathecal methotrexate 12mg/cytarabine 40mg/hydrocortisone 15mg (cycle 6)

↓

**Maintenance phase**

72 weeks; 24 3-week cycles

- Imatinib 400mg PO daily (continuous)
- Vinblastine 10mg IV (day 1)
- 6-mercaptopurine 50mg/m$^2$ PO (day 1-14)
- Dexamethasone 6mg PO BID (days 1-5)
- Methotrexate 30mg/m$^2$ PO (days 1, 8, 15)
- Intrathecal methotrexate 12mg/cytarabine 40mg/hydrocortisone (cycles, 5, 11, 17 & 23)

## Table S1. 48-month OS and RFS according to Year of Induction stratified by alloSCT in CR1

| Groups | Number of patients | 48-month OS rate (%) | 48-month RFS rate (%) |
|---|---|---|---|
| **2001-2009 and HCT at CR1** | 22 | 50.0 (95% CI, 28.2-58.4) | 50.0 (95% CI, 28.2-58.4) |
| **2001-2009 and no HCT at CR1** | 28 | 39.4 (95% CI, 21.1-57.3) | 35.8 (95% CI, 18.3-53.7) |
| **2010-2015 and HCT at CR1** | 23 | 38.3 (95% CI, 18.9-57.4) | 38.6 (95% CI, 19.3-57.7) |
| **2010-2015 and no HCT at CR1** | 35 | 66.5 (95% CI, 47.6-79.9) | 52.3 (95% CI, 34.3-67.5) |
| **2016-2019 and HCT at CR1** | 8 | 37.5 (95% CI, 8.7-67.4) | 37.5 (95% CI, 8.7-67.4) |
| **2016-2019 and no HCT at CR1** | 23 | 87.0 (95% CI, 64.8-95.6) | 69.3 (95% CI, 46.1-84.0) |
| | | p=0.0143 | p=0.1888 |

## Table S2. Characteristics of patients who underwent alloSCT in CR1 and those who did not.

| **Parameter** | **All Patients N=141** | **AlloSCT in CR1 N=53** | **No AlloSCT in CR1 N=88** |
|---|---|---|---|
| Median age, year (range) | 50.00 (18.0-79.1) | 46.00 (18.0-64.6) | 55.00 (24.0-79.1) |
| Age ≥60, n (%) | 39 (27.66) | 4 (7.55) | 35 (39.77) |
| Male, n (%) | 77 (54.61) | 30 (56.60) | 47 (53.41) |
| Female, n (%) | 64 (45.39) | 23 (43.40) | 41 (46.59) |
| Median WBC count, $x10^9$/L (range) | 19.20 (0.80-272.0) | 22.00 (0.80-230.0) | 16.00 (0.9-272.0) |
| WBC count, ≥30 $x10^9$/L (%) | 45 (34.35) | 18.00 (37.50) | 27.00 (32.53) |
| Extramedullary disease, n (%) | 14 (9.93) | 7 (13.21) | 7 (7.95) |
| CNS involvement at diagnosis, n (%) | 15 (10.64) | 7 (13.21) | 8 (9.09) |
| Prior cancer history, n (%) | 13 (9.63) | 4 (7.84) | 9 (10.71) |
| **Chemotherapy, n (%)** | | | |
| Pediatric-inspired multiagent protocol | 140 (99.29) | 53 (100.00) | 87 (98.86) |
| Other | 1 (0.71) | 0 (0.00) | 1 (1.14) |
| Asparaginase, n (%) | 35 (24.82) | 17 (32.08) | 18 (20.45) |
| **TKI, n (%)** | | | |
| Imatinib | 138 (98.57) | 51 (98.08) | 87 (98.86) |
| Dasatinib | 2 (1.43) | 1 (1.92) | 1 (1.14) |
| **Transplant in CR1, n (%)** | 53 (37.59) | 53 (100.00) | 0 (0.00) |
